# Validation of fracture-derived polygenic scores with FRAX for fracture risk prediction in postmenopausal women

**DOI:** 10.1007/s11657-026-01740-7

**Published:** 2026-07-27

**Authors:** Anqi Liu, Jianing Liu, Qing Wu

**Affiliations:** Department of Biomedical Informatics, College of Medicine, The Ohio State University, 250 Lincoln Tower, 1800 Cannon Drive, Columbus, OH 43210 USA

**Keywords:** Fracture risk, Polygenic risk score, Bayesian analysis, Osteoporosis, Genome-wide association study, Post-menopause, Genetic predisposition to disease, Risk assessment, Medical decision-making

## Abstract

***Summary*:**

We evaluated whether adding a genetic risk score derived from forearm fracture improves fracture prediction beyond standard clinical tools. Incorporation of GPS resulted in a small increase in time-dependent AUC, with improvement in NRI. Decision curve analysis suggested potential clinical benefit across certain threshold ranges.

**Background:**

Previous studies have incorporated estimated bone mineral density (eBMD)-derived polygenic scores into FRAX to improve fracture risk prediction, indirectly capturing genetic susceptibility through bone mineral density. However, the genetic architectures of fracture and BMD only partially overlap, suggesting that fracture-specific genetic risk may provide complementary and more direct biological information. With the availability of recently released forearm fracture GWAS summary statistics, we developed Bayesian genome-wide polygenic scores (GPS) derived from fracture risk and evaluated whether integrating fracture-derived Bayesian GPS into FRAX could enhance clinical fracture risk prediction.

**Methods:**

We constructed two Bayesian GPS using recently released UK Biobank forearm fracture GWAS summary statistics (GWAS Catalog study ID: GCST90281273) with two Bayesian frameworks: polygenic risk score-continuous shrinkage (PRS-CS) and summary-data-based Bayesian regression with continuous shrinkage and functional annotation integration (SBayesRC). The fracture-derived Bayesian GPS were integrated into FRAX to derive fracture-specific GPS-FRAX models. Model performance was evaluated in 10,135 postmenopausal women from the Women’s Health Initiative (WHI) using time-dependent area under the receiver operating characteristic curve (AUC), Brier score, net reclassification improvement (NRI), calibration analysis, and decision curve analysis (DCA).

**Results:**

Compared with the FRAX-CRF model (time-dependent AUC = 0.683), fracture-derived Bayesian GPS-FRAX models demonstrated modest improvements in discrimination, with time-dependent AUCs of 0.693 for PRS-CS and 0.690 for SBayesRC. Overall reclassification proportions were low (1.58% for SBayesRC and 1.82% for PRS-CS), but NRI was significantly improved, with overall NRI estimates of 2.20% (95% CI, 0.83 to 3.58%) for SBayesRC and 2.72% (95% CI, 1.34 to 4.19%) for PRS-CS. These findings indicate incremental predictive gain beyond clinical FRAX factors despite modest increases in discrimination.

**Conclusions:**

Incorporation of fracture-derived Bayesian GPS into FRAX resulted in a modest but statistically significant improvement in model discrimination, as reflected by a small increase in AUC. In addition, improvements in NRI and higher estimated net benefit in decision curve analysis suggest incremental clinical utility. However, the overall magnitude of improvement remained limited, indicating that the added predictive value beyond established clinical risk factors is modest. Further evaluation in more diverse populations is warranted.

**Supplementary Information:**

The online version contains supplementary material available at 10.1007/s11657-026-01740-7.

## Introduction

Osteoporosis, defined by decreased bone density and deterioration of skeletal microarchitecture, has become an escalating global health concern driven by population aging and the increasing prevalence of fragility fractures. In the USA, an estimated 10 million adults aged 50 years or older are affected by osteoporosis, while another 34 million are at increased risk, leading to nearly 1.5 million osteoporotic fractures each year [1]. These fractures substantially increase morbidity and mortality, result in considerable healthcare costs, and severely diminish patients’ quality of life [2]. Improving the accuracy of fracture risk prediction tools remains important for improving identification of individuals at elevated fracture risk.

Genetic factors play a substantial role in osteoporosis, with heritability estimates ranging from approximately 50 to 80% [3–7]. The exclusion of genetic information from current risk assessment models likely contributes to misclassification of individual risk and uncertainty in treatment decision-making. These limitations are particularly consequential among postmenopausal women, who often fall within intermediate or “gray zone” risk categories where clinical thresholds for intervention remain ambiguous [8–10].

Polygenic risk prediction using GWAS summary statistics has been widely applied across multiple complex diseases and traits, including cardiovascular disease, psychiatric disorders, and osteoporosis. Methodological frameworks [11] have demonstrated the scalability and practical utility of summary-statistics-based PRS approaches. Traditional clumping-and-thresholding (C + T) approaches are computationally efficient and widely used; however, they may incompletely account for linkage disequilibrium (LD) structure and highly polygenic genetic architectures. More recent Bayesian shrinkage methods, including polygenic risk score–continuous shrinkage (PRS-CS) and summary-data-based Bayesian regression with continuous shrinkage and functional annotation integration (SBayesRC), jointly model genome-wide SNP effects while accounting for LD structure, thereby improving effect size estimation and predictive performance for highly polygenic traits. In addition, SBayesRC incorporates functional genomic annotations, which may further enhance biological prioritization and prediction accuracy. Nevertheless, predictive performance remains influenced by factors such as ancestry matching, GWAS sample size, phenotype definition, and the transferability of effect estimates across populations.

Previous efforts [12–15] in osteoporosis have developed genetic risk scores primarily based on GWAS of BMD, estimated BMD (eBMD), or heel quantitative speed of sound (SOS). These approaches have generally demonstrated modest improvements in discrimination when incorporated into fracture risk prediction models, as reflected by small increases in AUC. However, as has been noted [16, 17], these phenotypes are not equivalent to fracture outcomes, and their use may introduce measurement-related limitations that reduce predictive relevance for actual fracture risk.

To address these clinical limitations, we developed two fracture-derived GPS based on newly released summary statistics from the forearm fracture GWAS and integrated them into the FRAX, hereafter referred to as GPS-FRAX. Specifically, we employed PRS-CS and SBayesRC, both of which model complex genetic architectures and account for linkage disequilibrium, with SBayesRC further incorporating functional genomic annotations. Our primary objective was to determine whether integrating fracture-derived Bayesian GPS into FRAX could improve individualized fracture risk stratification beyond FRAX alone.

## Materials and methods

### Study design and population

**Fig. 1:**
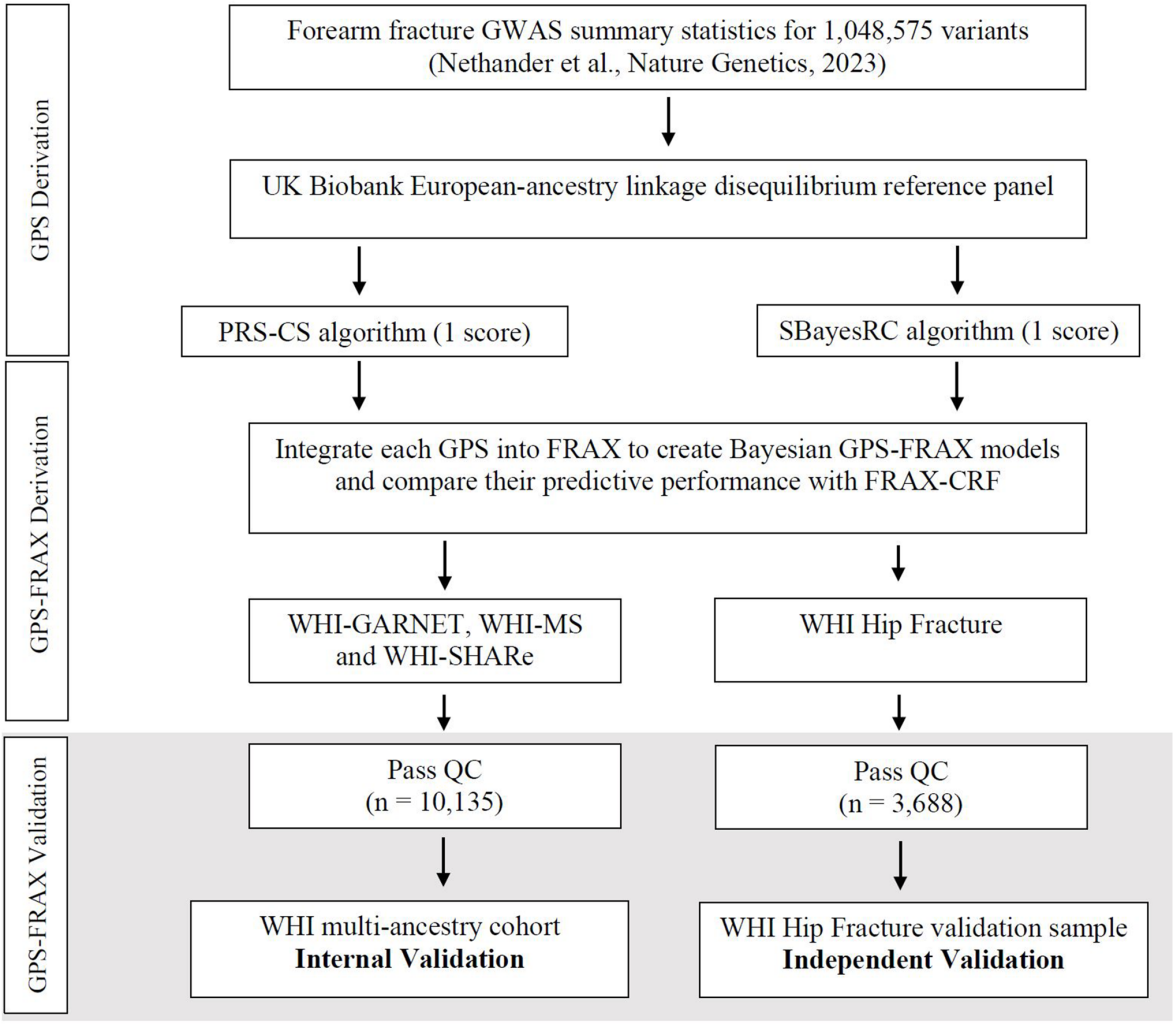
Workflow for fracture-derived Bayesian GPS Derivation, Validation, and Integration into FRAX. Genome-wide polygenic scores were derived from 1,048,575 variants using forearm fracture GWAS summary statistics (Nethander et al.). PRS-CS (continuous shrinkage Bayesian priors) and SBayesRC (summary-data-based Bayesian regression with functional annotation) were applied to construct two fracture-derived Bayesian GPSs. The GPSs were integrated into FRAX to develop Bayesian GPS-FRAX models, which were evaluated across WHI genomic sub-studies used for model development and internal validation. Independent validation was subsequently performed in the non-overlapping WHI Hip Fracture sub-study. Key QC criteria: genotype call rate ≥ 95%, Hardy-Weinberg equilibrium *P* ≥ 1 × 10^−6^, minor allele frequency ≥ 0.01. Abbreviations: GPS, genome-wide polygenic score; SNP, single-nucleotide polymorphism; GWAS, genome-wide association study; PRS-CS, polygenic risk score continuous shrinkage; SBayesRC, summary-data-based Bayesian regression with functional annotation; WHI, Women’s Health Initiative; FRAX, fracture risk assessment tool; MOF, major osteoporotic fracture

This retrospective cohort study used clinical and genetic data from postmenopausal women enrolled in the Women’s Health Initiative (WHI). Model development and internal validation were conducted using participants from the Genomics and Randomized Trials Network (WHI-GARNET), the WHI Memory Study (WHI-MS), and the SNP Health Association Resource (WHI-SHARe). Independent validation was performed in the WHI Hip Fracture GWAS sub-study (WHI-Hip Fracture). Data were obtained through the Database of Genotypes and Phenotypes (dbGaP; accession number phs000200.v12.p3). Participants were included if they had available genetic data, complete clinical variables, and documented follow-up fracture outcomes. Individuals with missing values in any clinical variables used for model construction were excluded. As a result, the analytic cohort represents a subset of WHI participants with complete genetic and phenotypic data, rather than the full WHI population**.** The final analytic cohort consisted of 10,135 postmenopausal women (Fig. 1).

### Fracture ascertainment and clinical data

Fracture outcomes included major osteoporotic fractures (MOF), defined as fractures of the hip, spine, wrist, or proximal humerus. Fracture events were identified through participant self-reports, which had been previously validated within the WHI cohort [18]. Participants were followed for an average of 16.4 years from baseline. For analysis, outcomes were restricted to a 10-year time horizon to align with the FRAX prediction framework. The endpoint was incident MOF within 10 years of baseline. Death before fracture was treated as a competing event.

### Genotype data and quality control

Genotyping was conducted using Illumina and Affymetrix platforms, with imputation using the 1000 Genomes Project (Phase 3 European reference panel) [19]. Quality control included sample call rates ≥ 95%, SNP call rates ≥ 90%, Hardy-Weinberg equilibrium (*P* ≥ 1 × 10⁻⁶), and minor allele frequency (MAF ≥ 0.01). Genotype QC was conducted using PLINK [20].

### Genome-wide polygenic score (GPS) calculation

To calculate GPS, we utilized publicly available summary statistics from the forearm fracture GWAS from the GWAS Catalog (study ID: GCST90281273) [21]. This GWAS analyzed genetic determinants of forearm fracture in the UK Biobank, providing large-scale summary data suitable for polygenic modeling. Forearm fracture is clinically relevant because it is a core component of MOF and strongly predicts future osteoporotic fractures [22]. The discovery dataset comprised 1,048,575 SNPs from the Forearm fracture GWAS. The final set of SNPs included in the GPS calculation depended on their availability and overlap with the WHI. GPS were calculated using two Bayesian methods: PRS-CS and SBayesRC. PRS-CS employs a continuous shrinkage prior on SNP effect sizes, incorporating linkage disequilibrium (LD) structure from a UK Biobank European reference panel, robustly capturing fine-scale LD patterns and improving predictive accuracy [23].

1 $$y=X\beta+\in,\in\sim\mathcal N\left(0,\sigma^2I\right)\,{GPS}_{PRS-CS,i}=\sum\nolimits_{j=1}^M\;{\widehat\beta}_j\;G_{ij}$$

2 $$\widehat{{\beta}_{j}}\sim \mathcal{N}\left(0,\phi {\psi}_{j}\right),{\psi}_{j}\sim Gamma\left(a,b\right)$$

PRS-CS estimates SNP effect sizes using a continuous shrinkage prior that integrates a global shrinkage parameter ($\phi$) and local (${\psi}_{j}$) shrinkage parameters to flexibly model varying degrees of sparsity and polygenicity. Posterior effects $\widehat{{\beta}_{j}}$ are inferred through a fully Bayesian Gibbs sampling algorithm, leveraging the UK Biobank European LD reference panel to account for linkage disequilibrium structure.

In SBayesRC, SNP effect sizes ${\upbeta}_{j}$ are modeled using an annotation-modulated mixture prior that extends the SBayesR framework by integrating functional annotations [24]. The model allows the prior variance of each SNP effect to depend on its functional annotation vector ${a}_{j}$, such that:

$${\upbeta}_{j}\sim \sum_{k=1}^{K}{\pi}_{jk}N(0,{\gamma}_{k}{\sigma}_{g}^{2})$$

$$f\left({\pi}_{jk}\right)= {\mu}_{k}+\sum_{l=1}^{c}{A}_{jl}{a}_{kl}$$

where ${\sigma}_{g}^{2}$ represents the total SNP-based genetic variance and ${\gamma}_{k}$ are scaling factors that determine the variance of each mixture component, and $f(\cdot )$ denotes a link function that maps the probability variable ${\pi}_{jk}$ to the real line. This formulation enables annotation-informed shrinkage, improving the estimation of causal effect sizes while accounting for LD and polygenic complexity.

Both PRS-CS and SBayesRC are fully Bayesian and tuning-free, requiring no manual selection of hyperparameters, as all shrinkage parameters are estimated directly from the summary statistics and LD reference panel. GPS values were Min-Max normalized to the 0–1 range.

### Fracture-derived Bayesian GPS-FRAX model development

In the Bayesian fracture-derived GPS-FRAX framework, GPS were integrated into FRAX using the 10-year MOF probability estimated by the FRAX tool based on clinical risk factors alone (FRAX-CRF) as the baseline predictor. FRAX-CRF denotes the FRAX-derived 10-year fracture probability obtained directly from the FRAX, which contains clinical risk factors including age, sex, body mass index (BMI), prior fracture, parental history of hip fracture, current smoking, alcohol use, glucocorticoid exposure, rheumatoid arthritis, and secondary osteoporosis, without inclusion of BMD or genetic information. To minimize confounding due to population stratification, we included the first ten principal components (PCs) of genetic ancestry, computed using EIGENSTRAT, as covariates in all GPS-adjusted models. The $P_{FRAX}$ be the 10-year FRAX major osteoporotic fracture probability. To account for the competing risk of death, we fitted a Fine-Gray subdistribution hazard model. Let$h^\ast\left(t/X\right)$ denote the subdistribution hazard for major osteoporotic fracture. The Bayesian GPS-FRAX model is specified as:

5 $$\mathrm{logh}^\ast(\mathrm t\vert\mathrm X)={\mathrm{logh}}_0(\mathrm t)+{\mathrm\beta}_1\ast\log\left(\frac{{\mathrm P}_{\mathrm{FRAX}}}{1-{\mathrm P}_{\mathrm{FRAX}}}\right)+{\mathrm\beta}_2\cdot\mathrm{GPS}+{\mathrm\beta}_3\cdot\mathrm{Age}+{\mathrm\beta}_4\cdot\left(\mathrm{GPS}\cdot\mathrm{Age}\right)+\sum_{\mathrm k=1}^{10}{\mathrm r}_{\mathrm k}{\mathrm{PC}}_{\mathrm k}$$

As the FRAX score is a bounded probability (0–1), its direct inclusion as a linear predictor in the Fine–Gray subdistribution hazard model may violate the linearity assumption. To address this, we applied a logit transformation to map the FRAX probability onto an unbounded scale. Following the analytic framework of the previous Bayesian GPS-FRAX study [12], age was retained as an independent covariate, and an interaction term (GPS × age) was included to account for potential age-dependent genetic effects on fracture risk. This specification allows the effect of polygenic risk to vary across age rather than assuming a constant genetic effect. Prior studies have suggested that genetic susceptibility to bone loss may change with age [25]. Consistent with this evidence, we incorporated the age × GPS interaction term to capture potential age-dependent heterogeneity in genetic effects. Separate models were developed using GPS derived from PRS-CS and SBayesRC.

Because the coefficient of the logit-transformed FRAX probability was estimated from the WHI cohort rather than fixed at 1, the primary GPS-FRAX models should be interpreted as recalibrated FRAX models augmented with genetic information. Accordingly, the primary analysis evaluates the incremental predictive value of GPS within a cohort-specific recalibration framework rather than treating FRAX as a fixed clinical risk score.

### Statistical analysis

Baseline characteristics were summarized as means with standard deviations (SD) for continuous variables and as frequencies with percentages for categorical variables. Between-group differences were assessed using chi-square tests, *t*-tests, or Fisher’s exact tests, as appropriate.

Participants were stratified into the top 5%, middle 90%, and bottom 5% of the fracture-derived GPS distribution. Differences in MOF risk across GPS strata were evaluated using Fine-Gray subdistribution hazard regression models, treating death as a competing event.

Discrimination was assessed using the time-dependent AUC at 10 years, and overall prediction accuracy was evaluated using the Brier score based on cumulative incidence function (CIF) predictions. Time-dependent AUC at year 10 was evaluated using stratified fivefold cross-validation. The dataset was randomly partitioned into five folds while preserving the proportion of fracture events in each fold. Four folds were used for model training and one fold for testing, iterating across all five partitions to ensure non-overlapping training and validation sets. The net reclassification improvement (NRI) and reclassification were assessed under the fixed 20% threshold based on the U.S. National Osteoporosis Foundation (NOF) [26].

Calibration at 10 years was evaluated under competing risk by comparing Fine-Gray-predicted CIFs with Aalen-Johansen observed risks across deciles of predicted risk. Calibration curves depict mean predicted versus observed 10-year risks within each decile; bootstrap bias-corrected curves were obtained to assess optimism.

Decision curve analysis was conducted to evaluate the clinical utility of the models across a range of threshold probabilities (0–25%) based on predicted 10-year cumulative incidence from Fine–Gray models.

To further evaluate the independent predictive contribution of fracture-derived GPS, we conducted a subgroup analysis among participants with available DXA measurements (*N* = 689). We compared the performance of four specific models: (1) FRAX-CRF (clinical risk factors only); (2) FRAX-BMD (clinical risk factors plus BMD); (3) FRAX-BMD + GPS; and (4) GPS + age + BMD. This comparison allows further validation of adding the GPS to the existing prediction model.

As a sensitivity analysis, we additionally evaluated offset-based Fine–Gray models in which the coefficient of the logit-transformed FRAX probability was fixed at 1 and treated as an offset term, while estimating only the contribution of GPS.

### Independent validation

Independent validation was conducted in a non-overlapping WHI sub-study (WHI-HIP), which did not include participants from the model development cohort. Time-dependent AUC and NRI were evaluated to assess whether the two GPSs improved predictive performance beyond FRAX.

### Software and statistical packages

All analyses utilized R software version 4.2.2 (R Foundation for Statistical Computing) [27]. We employed packages including “timeROC” version 0.4 (ROC analyses) [28] and “riskRegression” version 2026.02.13 [29] for this study.

### Ethical considerations and data availability

All analyses were conducted under Institutional Review Board approval from Ohio State University (IRB approval number 2022H0420). Genotype and phenotype data are available via dbGaP under approved access agreements.

## Results

### Study population characteristics

The analytic cohort comprised 10,135 postmenopausal women with a mean (SD) age of 64.3 (7.1) years, among whom 765 (7.55%) experienced a major osteoporotic fracture (MOF). During 10 years, 218 deaths without prior MOF occurred. Compared with women without fractures, those who sustained fractures were older (mean [SD]: 67.5 [6.6] vs. 64.1 [7.1] years; *P* < 0.01), had lower body weight (mean [SD]: 74.1 [15.3] vs. 77.8 [16.5] kg; *P* < 0.01), lower hip BMD (mean [SD]: 0.77 [0.12] vs. 0.88 [0.15] g/cm^2^; *P* < 0.01), and higher baseline FRAX-predicted 10-year MOF risk (mean [SD]: 13.4% [8.4] vs. 9.0% [6.7]; *P* < 0.01). The GPS was derived from forearm fracture GWAS summary statistics and, by design, represents a risk-increasing genetic profile, such that higher GPS values indicate greater susceptibility to fracture (Table 1).

**Table 1:** Baseline characteristics of 10,135 women in the WHI model-development and internal-validation cohort, stratified by MOF status

Characteristic	Participant cohort (218 deaths during follow-up)			
	without MOF (*n* = 9370)	with MOF (*n* = 765)	Overall (*n* = 10,135)	*P*-value^a^
Age (years), mean (SD)	64.05 (7.12)	67.49 (6.61)	64.31 (7.14)	< 0.01
Height (cm), mean (SD)	161.26 (6.13)	160.88 (6.32)	161.23 (6.14)	0.11
Race/Ethnicity				< 0.01
American Indian or Alaskan Native	26 (0.28%)	1 (0.13%)	27 (0.27%)	
Asian or Pacific Islander	57 (0.61%)	2 (0.26%)	59 (0.58%)	
Black/African American	2325 (24.81%)	59 (7.71%)	2384 (23.52%)	
Hispanic/Latino	1050 (11.21%)	64 (8.37%)	1114 (10.99%)	
White	5851 (62.44%)	635 (83.01%)	6486 (64.00%)	
Other	61 (0.65%)	4 (0.52%)	65 (0.64%)	
Weight (kg), mean (SD)	77.78 (16.49)	74.13 (15.25)	77.50 (16.42)	< 0.01
Hip BMD (g/cm^2^)^b^	0.88 (0.15)	0.77 (0.12)	0.87 (0.15)	< 0.01
GPS (PRS-CS), mean (SD)	0.50 (0.14)	0.52 (0.14)	0.50 (0.14)	< 0.01
GPS (SBayesRC), mean (SD)	0.51 (0.13)	0.52 (0.14)	0.51 (0.14)	0.11
Rheumatoid Arthritis, *n* (%)	469 (5.01%)	49 (6.41%)	518 (5.11%)	0.09
Parental fracture history, *n* (%)	2985 (31.86%)	310 (40.52%)	3295 (32.51%)	< 0.01
Glucocorticoid use, *n* (%)	2 (0.02%)	0 (0.00%)	2 (0.02%)	Not estimable
Previous osteoporosis, *n* (%)	474 (5.06%)	77 (10.07%)	551 (5.44%)	< 0.01
FRAX-predicted MOF risk, mean (SD)	9.03 (6.71)	13.36 (8.36)	9.36 (6.94)	< 0.01

Abbreviations: *SD* standard deviation, *GPS (PRS-CS)* genome-wide polygenic score derived by using polygenic risk score continuous shrinkage, *GPS (SBayesRC)*: genome-wide polygenic score derived by using summary-data-based Bayesian regression with functional annotation

^a^*P *value were obtained using *t*-tests for continuous variables and chi-square tests for categorical variables, except that Fisher’s exact test was used for glucocorticoid use

^b^Hip BMD available in a subset of participants (overall: *n* = 689, non-fracture: *n* = 647, fracture: *n* = 42)

### Crude 10-year cumulative incidence of MOF by GPS groups

**Fig. 2:**
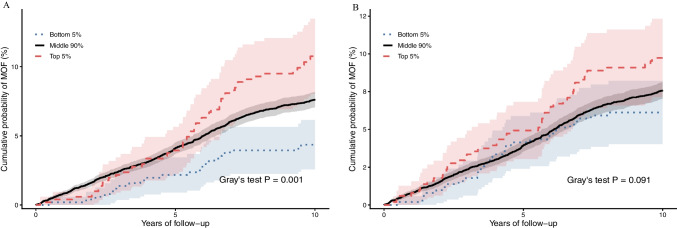
Cumulative incidence functions of major osteoporotic fracture (MOF) over 10 years according to extremes of fracture-derived genome-wide polygenic score (GPS), accounting for death as a competing risk. Participants were grouped into the top 5%, middle 90%, and bottom 5% of the GPS distribution. Shaded areas indicate 95% confidence intervals. **A** PRS-CS–derived GPS. **B** SBayesRC-derived GPS. *P*-values were obtained from Gray’s test comparing fracture risk across GPS strata

Figure 2 presents the crude 10-year CIF of MOF, accounting for the competing risk of death, stratified by extreme GPS groups (top 5% vs. bottom 5%). Visual separation between risk strata was more apparent for GPS (PRS-CS), whereas minimal separation was observed for GPS (SBayesRC). Differences in cumulative incidence across GPS strata were statistically significant for PRS-CS (*P* = 0.001), whereas no significant difference was observed for SBayesRC (*P* = 0.091).

### Model discrimination and robustness

The apparent time-dependent AUC for 10-year major osteoporotic fracture increased from 0.683 (95% CI, 0.664–0.701) with the FRAX-CRF model to 0.693 (95% CI, 0.674–0.711) with the GPS-FRAX (PRS-CS) model and 0.690 (95% CI, 0.671–0.710) with the GPS-FRAX (SBayesRC) model (Table 2). The improvement in AUC was statistically significant for both GPS-FRAX models compared with FRAX-CRF (PRS-CS: *P* = 0.004; SBayesRC: *P* = 0.026). Ten-year Brier scores were similar across models, decreasing from 0.0617 for FRAX-CRF to 0.0612 for GPS-FRAX (PRS-CS) and 0.0613 for GPS-FRAX (SBayesRC), suggesting minimal improvement in overall prediction error. In fivefold cross-validation, mean time-dependent AUC estimates were slightly lower (0.682 vs 0.6893 and 0.685), indicating minimal optimism and confirming the robustness of the observed discrimination improvement (Table S1).

**Table 2:** Apparent model discrimination for FRAX-CRF and fracture-derived GPS-FRAX models. Time-dependent AUCs at 10 years and Brier score were estimated under the competing-risk framework

Metric	AUC	Delta AUC	*P*-value	Brier score	Calibration slope
FRAX-CRF	0.6828 (0.6644, 0.7012)	Reference	Reference	0.0617 (0.0575, 0.0660)	0.9999
Bayesian GPS-FRAX(PRS-CS)	0.6926 (0.6740, 0.7112)	0.0098 (0.0030, 0.0166)	0.004	0.0612 (0.0570, 0.0654)	0.9581
Bayesian GPS-FRAX(SBayesRC)	0.6896 (0.6710, 0.7082)	0.0068 (0.0007, 0.0128)	0.026	0.0613 (0.0571, 0.0655)	0.9570

### Clinical reclassification and net reclassification improvement (NRI)

At the 20% National Osteoporosis Foundation (NOF) treatment threshold, incorporation of fracture-derived Bayesian GPS into FRAX resulted in minimal changes in clinical risk classification in the overall cohort. Overall reclassification rates were low for both models, with 1.58% of individuals reclassified using the GPS-FRAX (SBayesRC) model and 1.82% using the GPS-FRAX (PRS-CS) model (Table 3).

**Table 3:** Percentage reclassified and net reclassification improvement (NRI) for individual FRAX intervention criteria

Reclassification (%)	
All subjects (SBayesRC)	1.58%
All subjects (PRS-CS)	1.82%
Fracture outcome for NRI^**a**^ analysis (95% CI)	
NRI fracture, all subjects (SBayesRC)	2.48% (1.14%, 3.95%)
NRI fracture, all subjects (PRS-CS)	3.27% (1.95%, 4.71%)
NRI non-fracture, all subjects (SBayesRC)	−0.29% (−0.51%, −0.00%)
NRI non-fracture, all subjects (PRS-CS)	−0.54% (−0.79%, 0.30%)
NRI overall, all subjects (SBayesRC)	2.20% (0.83%, 3.58%)
NRI overall, all subjects (PRS-CS)	2.72% (1.34%, 4.19%)

^a^NRI was computed using standard 2 × 2 reclassification tables at the 20% threshold based on predicted 10-year fracture risk from competing-risk models accounting for death as a competing event

NRI analyses indicated improvement in overall risk reclassification. The GPS-FRAX (PRS-CS) model showed improvement in overall reclassification performance (overall NRI = 2.72%, 95% CI: 1.34–4.19%), while the GPS-FRAX (SBayesRC) model similarly improved classification accuracy (overall NRI = 2.20%, 95% CI: 0.83–3.58%). Event-specific analyses indicated improved reclassification among fracture cases, whereas the non-fracture NRI estimates showed no improvement for either model (Table 3).

### Calibration at 10 years under competing risk

**Fig. 3:**
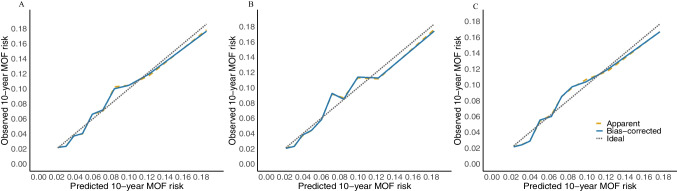
Calibration at 10 years under competing risk. Predicted 10-year MOF risks are grouped into deciles based on Fine–Gray predicted cumulative incidence. Points represent mean predicted risk within each decile, and observed risks are estimated using the Aalen–Johansen estimator within the same deciles. **A** GPS-FRAX(PRS-CS). **B** GPS-FRAX(SBayesRC). **C** FRAX-CRF

All three models demonstrated broadly similar calibration across the observed risk range, with apparent calibration curves closely tracking the ideal line (Fig. 3). Bootstrap bias-corrected curves overlapped with the apparent curves, indicating minimal optimism from internal model updating. Neither PRS-CS nor SBayesRC augmentation materially altered overall calibration relative to the FRAX-CRF baseline model.

### Clinical utility analysis

**Fig. 4:**
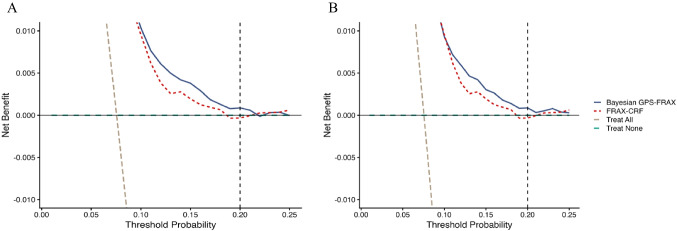
Decision curve analysis comparing the clinical utility of the FRAX-CRF model and the Bayesian GPS-FRAX model for predicting 10-year MOF risk. Net benefit was evaluated across a range of threshold probabilities (0–25%), accounting for death as a competing risk through Fine–Gray models. The Bayesian GPS-FRAX model demonstrated improved net benefit compared with the FRAX-CRF model across a range of clinically relevant thresholds. The vertical dashed line indicates the 20% intervention threshold. **A** GPS-FRAX (PRS-CS). **B** GPS-FRAX (SBayesRC)

DCA showed that Bayesian GPS-FRAX models provided a consistently higher net benefit than the FRAX-CRF model across clinically relevant thresholds (Fig. 4), particularly between 10 and 20%. However, the magnitude of improvement at the 20% treatment threshold was modest, consistent with the small but positive gains observed in discrimination and reclassification.

### Subgroup analysis with BMD integration

In the subset of participants with baseline BMD data, we evaluated the incremental value of GPS when combined with BMD. As shown in Table 4, the inclusion of GPS led to improved time-dependent AUC for both FRAX-CRF and FRAX-BMD models. Model 3 (FRAX-BMD + GPS) demonstrated the highest discrimination (AUC = 0.757 for PRS-CS). Notably, Model 4 (GPS + age + BMD), which excludes several clinical risk factors included in FRAX, showed comparable performance (AUC = 0.752 for PRS-CS) to Model 3. This finding suggests that fracture-derived GPS captures substantial risk information that complements BMD and age.

**Table 4:** Predictive performance of fracture-derived GPS integrated with BMD and clinical risk factors in the DXA-measured subset (*N* = 689)

	Time-dependent AUC
FRAX-CRF	0.7150 (0.6473, 0.7826)
FRAX-BMD	0.7340 (0.6667, 0.8010)
FRAX-BMD + GPS(PRS-CS)	0.7567 (0.6960, 0.8175)
FRAX-BMD + GPS(SBayesRC)	0.7541 (0.6933, 0.8148)
GPS(PRS-CS) + age + BMD	0.7516 (0.6869, 0.8162)
GPS(SBayesRC) + age + BMD	0.7478 (0.6831, 0.8125)

### Sensitivity analysis with fixed FRAX coefficient

To evaluate whether the observed improvement in predictive performance was driven primarily by recalibration of the FRAX predictor, we conducted an additional sensitivity analysis using offset-based Fine–Gray models in which the logit-transformed FRAX probability was included as an offset term with a fixed coefficient of 1. Under this specification, incorporation of fracture-derived GPS remained associated with modest improvements in discrimination. The time-dependent AUC increased from 0.683 for the FRAX-CRF model to 0.689 for the offset-based GPS-FRAX (PRS-CS) model and to 0.685 for the offset-based GPS-FRAX (SBayesRC) model. Overall, the findings remained directionally consistent with the primary analysis, suggesting that the incremental predictive contribution of GPS was not solely attributable to recalibration of the FRAX predictor (Table S2).

### Independent validation

Incorporation of fracture-derived Bayesian GPSs improved model discrimination in the independent WHI-HIP Fracture sub-study (*n* = 3688), with the 10-year time-dependent AUC increasing from 0.65 to 0.67 for GPS-FRAX (SBayesRC) and to 0.68 for GPS-FRAX (PRS-CS) (Table 5). Overall NRIs were 0.21% for both models; however, the 95% CI excluded zero for PRS-CS (0.06% to 0.53%) but included zero for SBayesRC (−0.06% to 0.54%). Event NRI was positive for both models, whereas non-event NRI showed no improvement. These findings are consistent with internal validation results, which demonstrated improvements in overall time-dependent AUC and overall NRI, but not in NRI for non-fracture individuals.

**Table 5:** Summarized predictive performance for independent validation

Metrics	FRAX-CRF	GPS-FRAX (PRS-CS)	GPS-FRAX (SBayesRC)
AUC	0.65 (0.63, 0.67)	0.68 (0.66, 0.69)	0.67 (0.66, 0.69)
NRI (overall)		0.21% (0.06%, 0.53%)	0.21% (−0.06%, 0.54%)
NRI (event)		0.21% (0.05%, 0.48%)	0.21% (0.05%, 0.47%)
NRI (non-event)		0.00% (−0.22%, 0.22%)	0.00% (−0.22%, 0.22%)

## Discussion

GPS have emerged as a comprehensive and scalable framework for aggregating genome-wide susceptibility into statistically incremental risk measures. Previously, we developed and validated two eBMD-derived GPS, demonstrating that incorporating eBMD GPS into FRAX significantly improved fracture risk prediction, not only in terms of AUC but also in clinical reclassification metrics such as the NRI. In the current study, we developed and validated two new GPSs using GWAS summary statistics for forearm fracture and examined whether fracture-derived GPS could further improve predictive performance beyond FRAX.

Incorporation of fracture-derived GPS into FRAX resulted in modest but consistent improvements in model discrimination, as evidenced by small increases in time-dependent AUC and improved NRI in the derivation cohort. In the independent validation cohort, GPS integration similarly yielded a modest gain in AUC. The PRS-CS model showed a small positive overall NRI, whereas the SBayesRC overall-NRI CI included zero; therefore, evidence for improved reclassification was not consistent across both GPS methods.

Several factors may explain the more modest performance of fracture-derived GPS compared with previously reported eBMD-derived GPS. Fracture phenotypes represent biologically heterogeneous outcomes influenced not only by skeletal fragility but also by environmental exposures, trauma mechanisms, and fall risk. Consequently, GWAS of fracture outcomes, including forearm fracture, have identified substantially fewer genome-wide significant loci than eBMD GWAS, potentially limiting the predictive capacity of fracture-based genetic models.

Several limitations should be acknowledged. First, fracture outcomes were based on self-reported data, which may introduce recall bias or misclassification despite prior validation within the WHI cohort. Second, the analytic cohort was restricted to participants with complete genetic and clinical data, which may introduce selection bias if missingness is related to specific underlying participant characteristics. Therefore, the study population represents a selected subset of WHI participants, and caution is warranted when generalizing these findings. Furthermore, hip BMD was available only in a relatively small subset of participants, which may introduce selection bias and limit representativeness. Therefore, analyses involving BMD should be interpreted with caution. As the primary analyses were based on the FRAX-CRF model, which does not require BMD, the main findings of this study were not affected by missing BMD data. In addition, although an independent validation was conducted using a non-overlapping WHI sub-study, it does not represent external validation in a distinct population. Third, the ancestry homogeneity of both GWAS discovery and validation datasets may limit generalizability to more diverse populations. Fourth, reliance on GWAS summary statistics derived from a single fracture site (forearm fracture) may introduce phenotype mismatch with the broader MOF outcome, potentially attenuating predictive performance and limiting the transferability of genetic effects. Finally, an additional methodological consideration relates to the treatment of FRAX in the primary GPS-FRAX models. Because the coefficient of the logit-transformed FRAX probability was estimated from the study cohort rather than fixed at unity, the primary models implicitly recalibrated FRAX to the WHI population before incorporating GPS. Consequently, the observed improvements in predictive performance reflect the combined effects of cohort-specific FRAX recalibration and genetic augmentation. Although sensitivity analyses using offset-based Fine–Gray models with a fixed FRAX coefficient yielded directionally similar results, the extent to which model improvement is attributable to recalibration versus genetic information cannot be completely disentangled.

Future research should aim to prospectively validate the fracture-derived Bayesian GPS across multi-ethnic cohorts and real-world clinical environments. Expanding analyses to include multi-site fracture GWAS and more comprehensive functional annotations may further enhance predictive performance and generalizability. Moreover, health-economic and implementation studies are warranted to evaluate the cost-effectiveness, real-world impact, and scalability of integrating genetic risk models into routine practice [30]. Collectively, these efforts will advance genetically informed precision fracture-risk assessment, enabling more personalized, equitable, and preventive strategies for osteoporosis management.

## Supplementary Information

Below is the link to the electronic supplementary material.

ESM 1 (17.7 KB)

## Data Availability

The individual-level data used in this study are available through controlled access from the database of Genotypes and Phenotypes (dbGaP) at https://www.ncbi.nlm.nih.gov/projects/gap/cgi-bin/study.cgi?study_id=phs000200.v12.p3. The genome-wide association summary statistics were obtained from the GWAS Catalog under study accession number GCST90281273 (https://www.ebi.ac.uk/gwas).
