# Supplementary material for: Validation of fracture-derived polygenic scores with FRAX for fracture risk prediction in postmenopausal women
Source: Arch Osteoporos. 2026 Jul 27;21(1):106. doi: 10.1007/s11657-026-01740-7 (PMC13408118; doi:10.1007/s11657-026-01740-7)
Supplement: Supplementary file 1 — (17.7 KB) [file 11657_2026_1740_MOESM1_ESM.docx]

**Supplemental Content**

Supplementary Table S1. Cross-validation of model discrimination

Supplementary Table S2. Sensitivity analysis using offset-based GPS-FRAX models with a fixed FRAX coefficient.

**Supplementary Table S1. Cross-validation of model discrimination.** Time- dependent AUC values for the FRAX-CRF model and GPS-FRAX models using polygenic scores derived through SBayesRC and PRS-CS. Results are shown across five folds and as an overall mean from 5-fold cross-validation.

| Fold | AUC(FRAX-CRF) | AUC(PRS-CS) | AUC(SBayesRC) |
| --- | --- | --- | --- |
| 1 | 0.672 | 0.668 | 0.679 |
| 2 | 0.657 | 0.702 | 0.693 |
| 3 | 0.675 | 0.663 | 0.660 |
| 4 | 0.705 | 0.701 | 0.688 |
| 5 | 0.701 | 0.690 | 0.692 |
| Mean | 0.682 | 0.685 | 0.683 |

**Supplementary Table S2. Sensitivity analysis using offset-based GPS-FRAX models with a fixed FRAX coefficient.**

| Metric | AUC | Brier Score | Calibration |
| --- | --- | --- | --- |
| FRAX-CRF | 0.6828(0.6644,0.7012) | 0.0617(0.0575,0.0660) | 0.9999 |
| Offset (FRAX)+GPS(PRS-CS) | 0.6894(0.6718,0.7071) | 0.0648(0.0604,0.0692) | 1.0336 |
| Offset (FRAX)+GPS(SBayesRC) | 0.6851(0.6675,0.7028) | 0.0651(0.0606,0.0694) | 1.1481 |
